# Improved Photocatalytic Activity of Dion–Jacobson-Type Tantalate Perovskites Modified with FeCl_2_

**DOI:** 10.3390/ma17194862

**Published:** 2024-10-02

**Authors:** Monica Pavel, Crina Anastasescu, Irina Atkinson, Florica Papa, Ioan Balint

**Affiliations:** “Ilie Murgulescu” Institute of Physical Chemistry of the Romanian Academy, 202 Spl. Independentei, 060021 Bucharest, Romania; canastasescu@icf.ro (C.A.); irinaatkinson@yahoo.com (I.A.); frusu@icf.ro (F.P.)

**Keywords:** oxide materials, molten salt route, optical properties, photocatalytic activity

## Abstract

A rapid and feasible approach was used to develop visible-light-driven-type Dion–Jacobson perovskites by the modification of the RbLaTa_2_O_7_ host (RbLTO) with FeCl_2_ through the molten salt route. X-ray diffraction (XRD) characterization showed that FeCl_2_-modified layered perovskite (e.g., Fe@RbLTO) preserved its lamellar structure. SEM micrographs confirmed the layered morphology of both RbLTO and Fe@RbLTO perovskite materials. The UV-Vis spectra illustrated a significant red shift of the absorption edge after Fe^2+^ modification, with the band gap energy reducing from 3.88 to 1.82 eV. H_2_-TPR measurements emphasized the anchorage of Fe^2+^ species located on the surface of the layered perovskite as well as in the interlayer space. The synthesized materials were valorized as photocatalysts for the degradation of phenol under both Xe lamp and simulated solar irradiation (SSL) conditions. The photocatalytic reaction follows first-order kinetics. By-product formations during phenol (Ph) degradation were identified and quantified using high-performance liquid chromatography (HPLC). Hydroquinone, 1,2-dihydroxi-benzene, benzoquinone, and pyrogallol were identified as the main Ph degradation intermediates. Pristine RbLaTa_2_O_7_ exhibited a phenol conversion value of about 17% using an Xe lamp, while a ≈ 11% conversion was achieved under SSL. A substantial increase in Ph conversion and selectivity was perceived after Fe^2+^ modification. Fe@RbLTO demonstrated superior photocatalytic performances (43% conversion of phenol under an Xe lamp, and 91% selectivity to aromatic intermediate compounds) at optimized reaction conditions. The stability of the Fe@RbLTO photocatalyst when exposed to an Xe lamp was also assessed. These results suggest that the existence of iron species on the layered perovskite’s surface is responsible for the improved redox properties of Fe@RbLTO, resulting in a valuable material for environmental applications.

## 1. Introduction

Large-scale pollution generated by human activities has stimulated interest in research communities to seek sustainable solutions to combat its adverse environmental effects. Although the prosperity brought by industrialization is a great success in terms of technology, it also led to a dramatic degradation of air, water, and soil quality. According to the World Health Organization (WHO), at least 1.7 billion worldwide people use drinking water that is contaminated due to the release of various organic contaminants [1]. The circular economy strategy and “green deal” imposed by international legislation enforce rapid actions to tackle pollution [2].

In response to these demands, photocatalysis is an effective process for various chemical transformations under ambient conditions to produce harmless end products. Moreover, the utilization of abundant solar energy is fascinating due to its green energy security and unmatched potential. This technique is also an alternative to conventional wastewater treatments that hold the advantages of being cheap, no secondary waste creation, and scale-up flexibility.

In recent years, material scientists have focused on designing efficient catalysts, optimizing synthesis parameters, fabricating diverse types of reactors, and understanding the reaction mechanism for visible-light-driven CO_2_-to-solar-fuel conversion [3]. As an example, the N-heterocyclic carbene (NHC)-ligated copper single-atom site (Cu SAS) embedded in a metal organic framework achieved a Faradaic efficiency of 81% for CO_2_ reduction to CH_4_ [4]. Furthermore, Zhang et al. [5] found that single-atom Ga catalysts (Ga SACs) can be employed as excellent CO_2_ reduction catalysts for CO products when the active center downsizes the atomic level. A novel tandem catalyst comprising an Ir single-atom (Ir_1_)-doped hybrid Cu_3_N/Cu_2_O multisite that operates efficiently when converting CO_2_ to CH_4_ has been reported by Chen and co-workers [6]. Additionally, the continuous growth of surface nanoengineering plays an important role in the catalysis area, with a promising outlook.

The photocatalytic removal of several organic and inorganic contaminants was recently reviewed [7], emphasizing the key factors contributing to their mineralization Among different aromatic compounds, phenol is a representative pollutant discharged from various industries, including coal gasification, oil refining, herbicide and fungicide production, detergent manufacturing, and cosmetic industries [8]. It is well documented that hydroxyl radicals are highly reactive entities [9] produced from two pathways: (i) O_2_ existing in water is reduced to •O_2_^−^, which then reacts with H^+^ to generate •OOH, followed by the decomposition to •OH radicals, and (ii) the oxidation of OH^−^ groups.

An ideal photocatalyst should meet certain requirements, such as a broad light-absorption range, band gap tunability, high density of reactive sites, redox potential, and effective charge carrier mobility [10]. Perovskite oxides possess unique electronic and optical properties, ideal for hydrogen production [11], the removal of pollutants [12], and CO_2_ conversion [13,14], as well as for applications as sensor materials, ferroelectrics, and dielectrics [15,16]. One of the most common types of layered oxides, Dion–Jacobson (DJ) perovskite has the general formula of M[A_n-1_B_n_O_3n+1_], where M is the interlayer alkali cation, A is the lanthanide cation surrounded by 12 oxygen atoms, B is the transition metal, and n describes the number of BO_6_ octahedra. As an example, the structure of RbLaTa_2_O_7_ is based on a double-layered perovskite-type structure made up of TaO_6_ sheets with La ions filling in the interstice a of TaO_6_ octahedron and the Rb^+^ cation located in the interlayer [17]. An essential feature of layered perovskites consists of their ability to accommodate a variety of spacers, providing a route to modify their chemical and physical properties through ion exchange [18], pillaring, exfoliation [19,20], or restacking methods [21,22]. Thus, novel layered compounds exhibiting new properties can be fabricated [23,24].

The initial research on layered tantalates, MLnTa_2_O_7_ (M = Cs, Rb, Na, H, Ln = La, Pr, Nd, and Sm), with a partly filled La 4f shell, was reported by Machida et al., showing their efficiency for overall water splitting under UV-light [25]. Proton-exchanged HLaNb_2_O_7_ also demonstrated an interesting photocatalytic performance for hydrogen production under UV-light exposure [26]. Nitrogen-doped layered perovskite CsM_2_Nb_3_O_10_ (M = Ba, Sr) showed significant activity for the photocatalytic degradation of methylene blue compared to its corresponding parent oxides [27].

Some issues of oxide materials obtained from conventional solid-state preparation are uncontrollable grain growth, the segregation of constituents, or the loss of the stoichiometry due to the volatilization of carbonate precursors. These issues result in poorer catalytic activity. Suitable synthesis routes in which new compounds or original morphologies are obtained with less time consumption and mild conditions are one of the most fruitful approaches. A simple topochemical route was employed by J.B. Wiley and collaborators [28,29,30] to prepare a series of (ACl)LaNb_2_O_7_-layered oxyhalides perovskites by exchanging alkaline ions from the ALaNb_2_O_7_ host (A = Li, Na, K, or Rb) with metal halides (MX)^+^ (M = Co^2+^, Cu^2+^, Mn^2+^, Ni^2+^, and X = Cl^−^, Br^−^). The resulting composites consist of MO_2_X_4_ octahedra that corner share with NbO_6_ octahedra from perovskite slabs. In the same manner, transition-metal-layered perovskites, A_0.5_LaNb_2_O_7_ (A = Fe, Ni, and Cu), were synthesized and thoroughly characterized [31]. Furthermore, Maggard et al. revealed that using low-melting inorganic salts as reaction media dramatically shortened the synthesis time of modified perovskites, leading to highly crystalline particles with nanometric sizes [32]. As an example, RbLaNb_2_O_7_ synthesized from RbCl-molten flux revealed high photocatalytic performances with an H_2_ generation rate in the range of 1457–2102 µmol·g^−1^·h^−1^ under UV light and 2–20 µmol·g^−1^·h^−1^ under visible-light exposure [33]. In another study, Porob and Maggard [34] demonstrated that the rapid molten salt route was effective to exchange copper chloride layers into an RbLaTa_2_O_7_ perovskite host producing (CuCl)^+^(LaTa_2_O_7_)^−^. The short reaction time (1 h) of this approach was achieved by the facile diffusion of Rb^+^ and CuCl^+^ species in the molten salts. Moreover, the intercalated (CuCl)^+^ layers added a new higher-energy valence band shifting the band gap of CuClLaTa_2_O_7_ to the visible region. However, the preliminary photocatalytic tests performed by the authors showed this compound had low activity. Also, (FeCl)LaNb_2_O_7_ was originally prepared by Viciu et al. [35] at a moderate temperature (350 °C), but with a longer reaction time (e.g., 14 days). Furthermore, the photocatalytic properties of (FeCl)LaNb_2_O_7_ remain unexplored.

In response to these findings, the molten flux approach was employed in the present study to develop a visible-light-driven catalyst through the FeCl_2_ modification of an RbLaTa_2_O_7_-layered perovskite. The novelty of this study provides a better understanding of the interaction between the incorporated FeCl_2_ and the RbLaTa_2_O_7_ host. Moreover, the influence of Fe species on the photocatalytic performance of Fe@RbLTO for the degradation of phenol under an Xe lamp and the simulated solar irradiation (SSL) were assessed and compared with that of the original RbLaTa_2_O_7_. The importance of the redox effect of the iron species in determining the activity and selectivity of the synthesized photocatalyst was demonstrated. To the best of our knowledge, this is the first report on the photocatalytic removal of phenol over Fe^2+^-modified RbLaTa_2_O_7_-layered perovskites.

## 2. Materials and Methods

### 2.1. Preparation of Photocatalysts

Firstly, the solid-state synthesis of the original layered tantalate, RbLaTa_2_O_7_ (denoted as RbLTO), was performed by combining stoichiometric quantities of Rb_2_CO_3_, La_2_O_3_, and Ta_2_O_5_ (with a 50% molar excess of Rb_2_CO_3_). The reactants were mixed together and placed in an alumina crucible, and calcined at 1200 °C in an air atmosphere for 18 h, with intermediate grinding. After cooling to room temperature, the white product was washed and then dried at 110 °C overnight.

The FeCl_2_-modified RbLaTa_2_O_7_ perovskite (designated as Fe@RbLTO) was created by using molten salts as the reaction media following a previously reported procedure [33]. The Fe^2+^-modified catalyst procedure was carried out in a homemade “U”-shaped quartz tube reactor (10 mm inner diameter, 1 mm wall thickness, and 140 mm height). During the synthesis procedure, the temperature was monitored with a vertical thermocouple placed inside the quartz reactor. The outlet of the reactor was isolated to reduce heat loss, and a continuous flow of 5% H_2_/Ar gas was passed through the reactor. The gas flow inlet was controlled by flow meters. In this way, the RbLaTa_2_O_7_ host reacted with anhydrous FeCl_2_ and molten KNO_3_ at a 1:2:20 molar ratio of layered perovskite–iron chloride–molten flux. The reactants were heated at 350 °C for 1 h in a 5% H_2_/Ar (90 mL·min^−1^) atmosphere. The obtained red product was washed with hot distilled water and dried overnight at 110 °C.

### 2.2. Structural Characterization

The crystalline structure of the powders was studied by X-ray diffraction (XRD) using a Rigaku Corporation Ultima IV diffractometer (Tokyo, Japan) with monochromatic Cu Kα radiation. The crystallite’s size was estimated using Sherrer’s equation. The morphology of the solids was analyzed using a Tescan Vega 3LMH scanning electron microscope (SEM) (Brno, Czech Republic). UV-Vis spectra were acquired using a Perkin Elmer Lambda 35 spectrophotometer (Shelton, CT, USA) equipped with an integrating sphere. Tauc plots were plotted as (F(R) × hν)^1/2^ versus hν (eV) for indirect band gap transitions from the inflection point tangent to the liner portion of the absorption curve. Diffuse reflectance infrared (DRIFT) spectra were acquired with a JASCO FT/IR-4700 spectrometer (Jasco, Tokyo, Japan) by the acquisition of 128 scans in the 4000–400 cm^−1^ wavelength domain. Attenuated total reflectance Fourier transformed infrared (ATR-FTIR) spectra were measured with the JASCO FT/IR-4700 spectrometer (Tokyo, Japan) equipped with a diamond crystal with a scanning speed of 128 scans/min, triangle apodization, and a resolution of 4 cm^−1^. The temperature-programmed reduction measurements (H_2_-TPR) were collected with a CHEMBET-3000 Quantachrome Instrument (Boynton Beach, FL, USA) equipped with a thermal conductivity detector (TCD). Typically, the fresh sample (0.03 g) was heated up to 800 °C at the constant rate of 10 °C·min^−1^ of the 5% H_2_/Ar (flow rate of 70 mL·min^−1^). H_2_ consumption was estimated from the area of the recorded peaks. The calibration of the TCD signal was performed by injecting a known quantity of hydrogen (50 µL) into the Ar carrier gas. The experimentally obtained peak surface (mV∙s) was converted into micromoles of hydrogen.

### 2.3. Photocatalytic Test and Analysis

The photocatalytic degradation of phenol was performed in a photoreactor thermostated at 18 °C. Irradiation was generated by a sunlight simulator (AM 1.5G, Peccell-L01, Yokohama, Japan) equipped with a 150 W short-arc Xe lamp (1000 W·m^−2^). The scheme of the experimental equipment was detailed in a previous work [36]. In a typical test, 110 mL of aqueous phenol solution (25 mg·L^−1^, pH = 6.5) and 0.050 g of photocatalyst were added into the quartz photoreactor under continuous stirring. The photoreactor was fitted with a quartz window measuring 4.5 × 4.5 cm^2^ for light irradiation. During the tests, Ar (10 mL·min^−1^) and O_2_ (1 mL·min^−1^) flows were continuously purged into the reaction vessel and passed through a refrigerant cooled to −5 °C, in order to prevent the liquid vapors entering into the GC equipment (Buck Scientific, Norwalk, CT, USA). To establish absorption–desorption equilibrium, before turning on the lamp, the samples were kept in the dark, under stirring, for 50 min.

The stability of Fe@RbLTO was monitored by testing the photocatalyst under the same conditions as the above-stated experiment (25 mg·L^−1^, pH 6.5, and 0.050 g of catalyst) under Ar and O_2_ flow conditions, first for 50 min in the dark, followed by 10 h of Xe light exposure).

The evolution of the liquid phase composition (phenol and intermediate ring compounds) was monitored by means of a high-performance liquid chromatograph (HPLC) (Waters, Alliance e2659, Milford, MA, USA) equipped with UV-Vis detector (λ = 273 nm) (Waters, model 2489), Kromasil 100 5-C18 column, mobile phase Milli-Q water:methanol = 50:50 (*v*/*v*), flow rate of 1 mL·min^−1^, analysis time of 30 min, column temperature of 35 °C, and injection volume of 2 µL. The evolved gases were analyzed every 30 min with an online gas chromatograph (Buck Scientific, Norwalk, CT, USA) equipped with a TCD detector. H_2_ and O_2_ were separated and quantified on a Molecular Sieve 5 Å column, whereas CO_2_ was monitored on a Haysep column. Superoxide (O_2_^−^) formation was monitored by exposing the catalyst to simulated solar light, according to the following parameters: 0.003 g of powder was suspended in an aqueous solution (3 mM) of 2,3-Bis(2-metoxy-4-nitro-5-sulphenyl)-2H-tetrazolium-5-carboxanilide, and the photogenerated O_2_^−^ reducing it and leading to XTT formazan formation. Accordingly, a broad peak located at 470 nm appeared when the UV-Vis spectra were recorded with an Analytik Jena Specord 200 Plus spectrophotometer (Jena, Germany).

## 3. Results

### 3.1. XRD Characterization of the Catalysts

**Figure 1:**
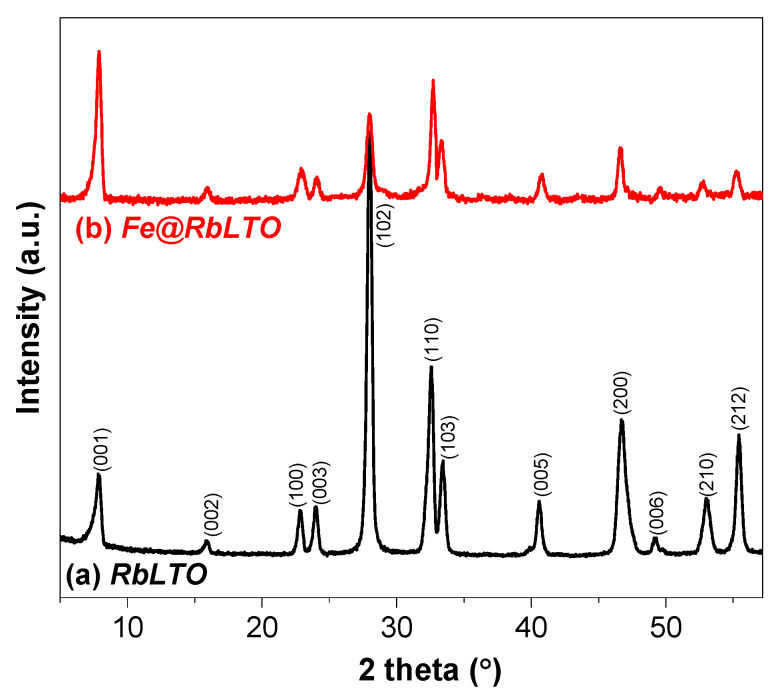
XRD pattern of the synthesized materials: (**a**) pristine RbLTO and (**b**) Fe@RbLTO-modified layered perovskite.

The XRD patterns (Figure 1) of the RbLTO solid exhibited a typical lamellar structure with intense reflections specific to RbLaTa_2_O_7_ Dion–Jacobson-layered perovskite materials [37]. The XRD patterns are indexed in the tetragonal structure of the spatial group *P4/mmm* (PDF card no. 01-089-0389). The Fe^2+^-modified perovskite (Fe@RbLTO solid) maintained the layered structure of the original tantalate perovskite. It is interesting to note that there are no reflections corresponding to iron oxide phases in the Fe@RbLTO sample. However, the structure of Fe@RbLTO may have small and well-dispersed iron oxide crystallites, which are under the XRD detection limit. The modification of the RbLTO perovskite with Fe ions caused a slight increase in the crystallite’s size, while the basal spacing of the (001) reflection of both samples did not vary noticeably. The corresponding lattice parameters, the *d*-spacing, and the crystallite sizes of the solids calculated by Scherer’s equation are presented in Table 1.

### 3.2. SEM Morphology of Catalysts

**Figure 2:**
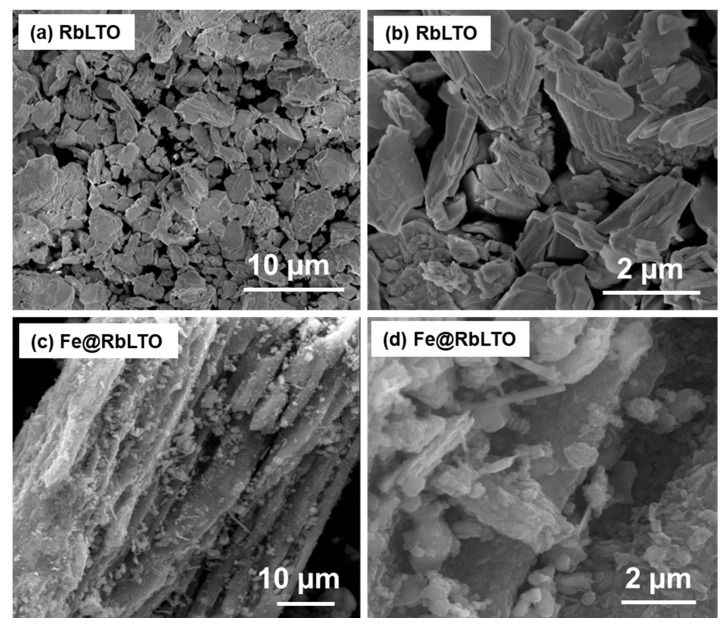
SEM images of (**a**,**b**) RbLTO and (**c**,**d**) Fe@RbLTO-modified catalyst.

The scanning electron microscopy (SEM) images of the RbLTO (Figure 2a,b) and Fe@RbLTO (Figure 2c,d) perovskites revealed a plate-like morphology and agglomerated particles for both samples. This observation is similar to other previously reported layered oxides [18].

**Figure 3:**
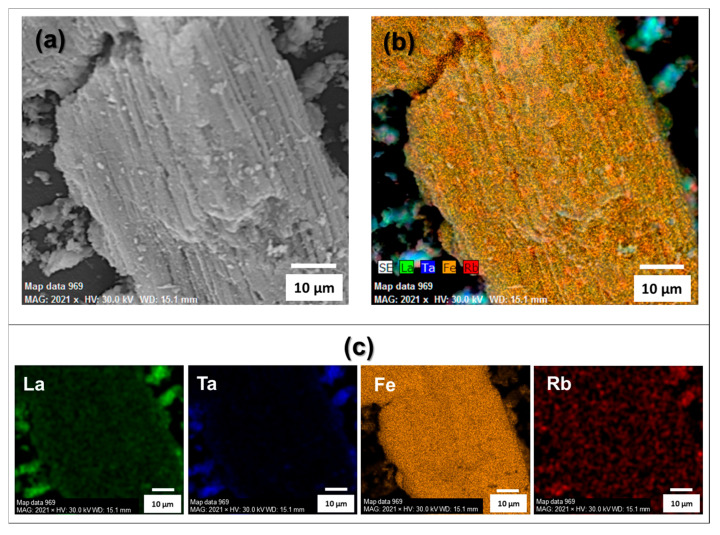

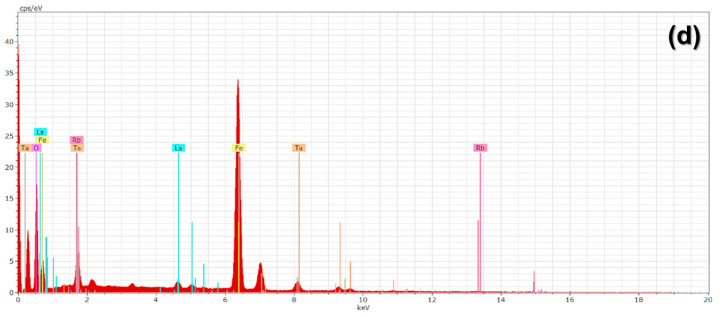
Fe@RbLTO-modified catalyst with its corresponding EDX mapping pictures (**a**–**d**).

Energy-dispersive X-ray spectroscopy (EDX) mapping of the Fe-modified layered perovskite (Figure 3a–d) reveals the uniform distribution of the elements Fe, Rb, La, and Ta.

### 3.3. UV-Vis Spectroscopy

**Figure 4:**
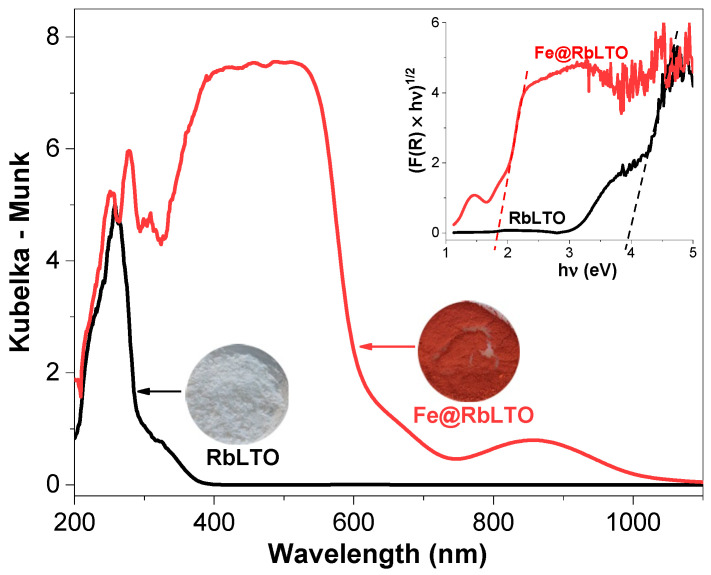
UV-Vis spectra of the pristine RbLTO and Fe@RbLTO-layered perovskites. The insert box shows the Tauc plots for the calculation of the band gap energies of both samples.

The optical properties of the pristine RbLTO and the modified Fe@RbLTO were investigated by UV-Vis spectroscopy (Figure 4). The white RbLTO powder exhibits strong absorption in the ultraviolet region (<380 nm) and no obvious absorption in the visible light region. The optical band gap energy (Eg) for this compound estimated by the Tauc curve (insert of Figure 4) was about 3.88 eV, being consistent with the literature results for tantalate compounds [25]. Conversely, the modification of the perovskite with FeCl_2_ caused the change in the product’s color from white to red. The absorption band edge of Fe@RbLTO extended toward the visible-light region (λ > 400 nm), and the red shift of the modified perovskite is attributed to the incorporation of Fe^2+^ species. An additional absorption band located at a wavelength higher than 740 nm was perceived for this sample and could be indicative of some different iron species while maintaining a layered lattice, as confirmed by the XRD analysis. Skvortsova et al. observed, for the natural beryl (Be_3_Al_2_Si_6_O_18_) spectra, a wide absorption band, with a maximum peak at 813 nm ascribed to the internal electron transition of ^5^T_2_ (^5^D) → ^5^E (^5^D) of Fe^2+^ ions localized in the octahedral aluminum sites of beryl [38].

The valence band of RbLaTa_2_O_7_ consists of O2*p* orbitals, whereas the conduction band is formed by Ta5*d* orbitals [39]. After FeCl_2_ modification, the *4s* orbital of Fe mixes with O2*p* orbital to present an upward shift of the valence band, leading to a narrow band gap. The calculated band gap energy of Fe@RbLTO was observed at about 1.82 eV. Thus, the reduced energy required for electron excitation is likely to improve the photocatalytic performances of the modified perovskite.

### 3.4. DRIFT and ATR Spectroscopies

**Figure 5:**
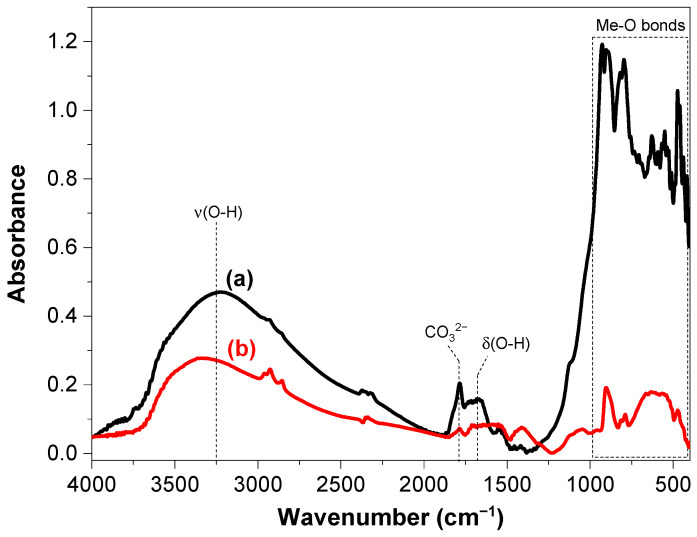
DRIFT spectra of (**a**) RbLTO and (**b**) Fe@RbLTO-layered perovskites.

The attenuated total reflectance (ATR) and diffuse reflectance infrared Fourier transforms spectroscopy (DRIFTS) techniques provide complementary information about the structure of the catalysts. The DRIFT spectra of the RbLTO solid (Figure 5) display a band at 3227 cm^−1^ assigned to the stretching vibration of OH groups, which shifted toward a larger value, namely 3356 cm^−1^, for the Fe@RbLTO catalyst. It is noteworthy that the band intensities of the Fe^2+^-containing layered perovskite decreased after iron species incorporation. The band at 1671 cm^−1^ for both samples is attributed to the bending vibration of the O-H group in adsorbed water molecules. The asymmetric stretching vibration of CO_3_^2−^ was observed at 1780 cm^−1^. The bands observed at a wavelength lower than 950 cm^−1^ are due to the metal–oxygen vibrations in both samples.

**Figure 6:**
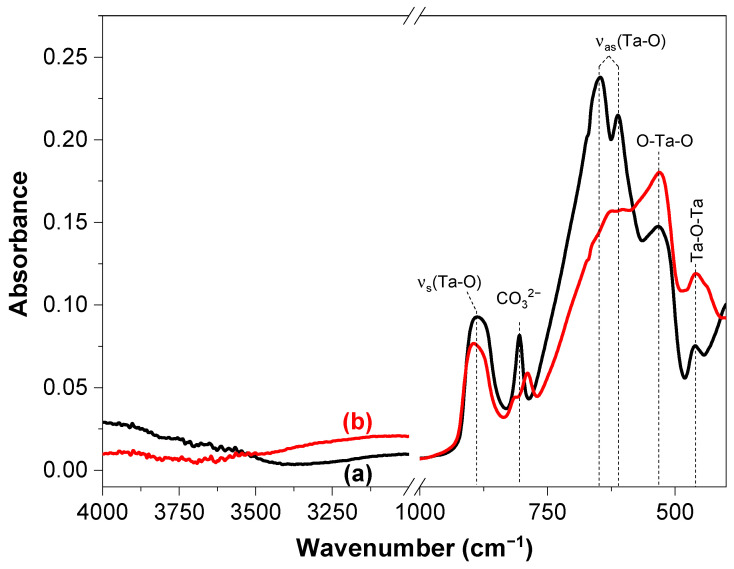
ATR spectra of (**a**) RbLTO and (**b**) Fe@RbLTO-layered perovskites.

Additionally, the ATR spectra provide valuable details on the effect of Fe^2+^ over the RbLaTa_2_O_7_ structure (Figure 6). Vibration modes appearing at 889 cm^−1^, 650 cm^−1^, and 608 cm^−1^ are associated with symmetric and asymmetric stretching vibrations of Ta-O. The bands at 533 cm^−1^ and 458 cm^−1^ are related to O-Ta-O and Ta-O-Ta bridges, respectively [40,41], while the band located around 809 cm^−1^ involves some carbonate species [42]. The modification of the layered perovskite with FeCl_2_ induces a slight shifts toward lower wavenumbers (<1000 cm^−1^) in the ATR spectra.

### 3.5. H_2_-TPR Measurements

**Figure 7:**
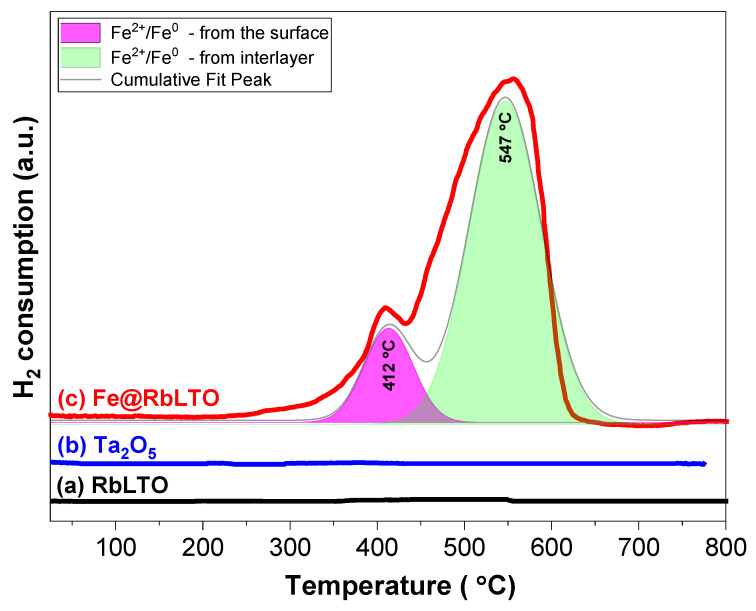
H_2_-TPR profiles of the (**a**) RbLTO, (**b**) Ta_2_O_5_, and (**c**) Fe@RbLTO solids.

To further confirm the successful modification of the layered tantalate perovskite with Fe^2+^ ions, the optical properties were corroborated with those of the thermo-programmed reduction measurements (H_2_-TPRs). The RbLTO (Figure 7a) solid shows no reduction peaks in the temperature range of 25–800 °C, confirming that Rb^+^ and La^3+^ species are not reducible, and Ta^5+^ is reduced at temperatures higher than 800 °C (Figure 7b) [41]. After the introduction of Fe species (Figure 7c), two regions of reduced temperatures were perceived, suggesting a multiple-step reduction. The peak occurring at temperatures below 500 °C (max. at ≈ 412 °C) is assigned to the reduction in the Fe^2+^/Fe^0^ species localized on the surface of the catalyst. In the region with temperatures higher than 500 °C (max. at ≈ 547 °C), the Fe^2+^/Fe^0^ species localized in-between the layers of the perovskite are reduced.

The calculated amount of hydrogen consumption (expressed in µmoles·g^−1^) by iron species localized on the surface of the Fe@RbLTO catalyst was about 228.63 µmoles·g^−1^, while, for the number of iron species situated in the perovskite’s interlayer, a higher H_2_ consumption value of 929.37 µmoles·g^−1^ was determined. In this way, the existence of Fe^2+^ on the surface as well as in-between the layers of the perovskite is confirmed. These results are in agreement with the SEM-EDX measurements of the Fe@RbLTO catalyst. It is noteworthy that X-ray diffraction is not a sufficiently sensitive tool to determine the structure of very-thin oxide layers or amorphous phases. Given the small amount of Fe^2+^ on the catalyst’s surface, this may be the reason why no diffraction lines originating from iron oxides were perceived on the XRD pattern of Fe@RbLTO.

### 3.6. Photocatalytic Degradation of Phenol

**Figure 8:**
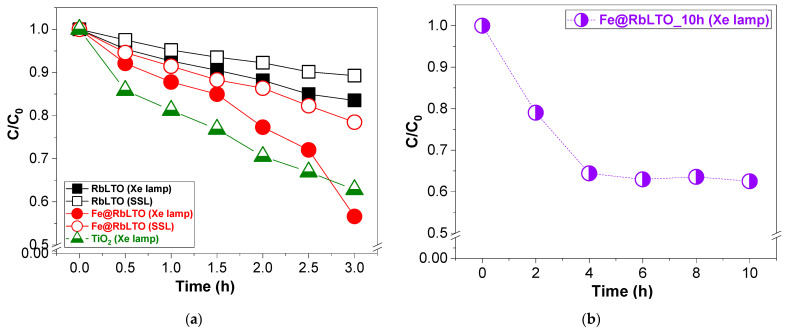
(**a**) Photocatalytic degradation curves for phenol photodegradation after 3 h of light exposure over the synthesized catalysts and TiO_2_ (Merck) reference; (**b**) stability test for the photodegradation of phenol over Fe@RbLTO after 10 h of Xe light irradiation. Reaction conditions: 0.050 g of catalyst, 25 mg·L^−1^ aq. phenol, pH 6.5, and T = 18 °C.

The photocatalytic activities of the original RbLTO and the Fe@RbLTO-modified catalysts were evaluated for the photocatalytic degradation of phenol under an Xe lamp and simulated solar irradiation (SSL). For comparison, TiO_2_ powder purchased from Merck (CAS no. 13463-67-7) was used as a reference and tested for an identical reaction under an Xe lamp. The time-dependent degradation curves of the above-stated photocatalysts are illustrated in Figure 8a. The results indicate that the Fe^2+^-modified perovskite exhibits higher photocatalytic activity for phenol photodegradation under Xe light exposure as compared to the original RbLTO catalyst. It should be noted that the activity of Fe@RbLTO starts slowly and increases rapidly after 2 h of reaction. This fact implies that the iron-modified photocatalyst needs time to activate oxygen. While the photocatalytic test is carried out under sunlight, the activity of both photocatalysts is slightly lower; however, the Fe@RbLTO catalyst always exhibits the highest activity in terms of phenol conversion related to the RbLTO unmodified catalyst. However, the TiO_2_ Merck reference showed higher photo-activity when degrading phenol under an Xe lamp compared to the synthesized perovskites. The stability test (Figure 8b) performed for Fe@RbLTO shows that the photocatalyst’s activity remained decent after 10 h of Xe light exposure.

The conversion of phenol (Table 2) to Fe@RbLTO reaches ≈ 43% after 3 h of Xe lamp exposure, being higher than that of unmodified RbLTO (≈17%) and the TiO_2_ reference (≈37%). On the other hand, when the photocatalytic activity is evaluated under simulated solar irradiation, both perovskite-based catalysts degraded phenol more slowly, with conversion values reaching about 22% for Fe@RbLTO and only ≈ 11% for RbLTO. Obviously, the modification of the RbLaTa_2_O_7_ perovskite with iron species influences phenol conversion and selectivity. The enhanced photo-activity of Fe@RbLTO compared to the original RbLTO catalyst is favored by its narrower band gap (1.88 eV), as determined by UV-Vis spectroscopy. Additionally, the H_2_-TPR result confirms the presence of Fe^2+^/Fe^0^ species localized on the photocatalyst’s surface, which are responsible for its higher activity.

The kinetics of phenol degradation follows the pseudo-first-order reaction, and the apparent rate constant k (min ^−1^) was determined from the slope of ln(C/C_0_) against time (t). C_0_ is the initial concentration of the pollutant and C is the concentration of the pollutant at time (t). The order of k values after 1.5 h of reaction under Xe lamp exposure follows the following sequence: 0.194 (TiO_2_) > 0.119 min^−1^ (Fe@RbLTO) > 0.066 min^−1^ (RbLTO). In the presence of solar light, the rate constant values are 0.078 min^−1^ for Fe@RbLTO and 0.041 min^−1^ for RbLTO. These results demonstrate that the presence of the Fe^2+^ species enhances the photodegradation of phenol over the modified photocatalyst.

**Figure 9:**
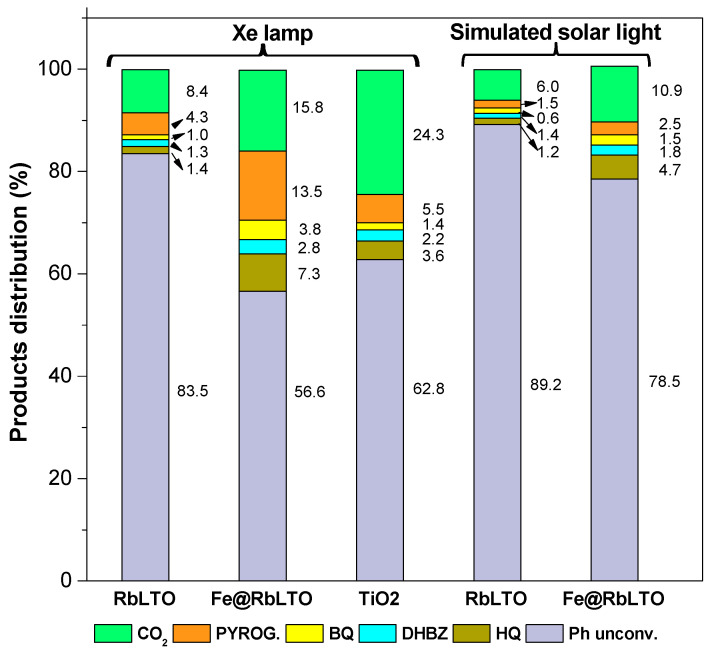
Product distribution (% µmoles) after 3 h of reaction over the RbLTO and Fe@RbLTO photocatalysts evaluated under both Xe lamp and simulated solar light conditions. TiO_2_ Merck is used as a reference and tested under an Xe lamp. Legend: Ph unconv = unconverted phenol, HQ = hydroquinone, DHBZ = 1,2-dihydroxi-benzene, BQ = benzoquinone, and PYROG = pyrogallol.

Figure 9 displays the distribution of the intermediate products at the end of the reaction along with unconverted phenol (expressed in µmoles %). Upon the Xe light exposure of the RbLTO, Fe@RbLTO, and TiO_2_ references, phenol photo-oxidation produces aromatic intermediates, including hydroquinone (HQ), 1,2 di-hydroxy-benzene (DHBZ), 1,2,3-benzene-triol (pyrogallol), and benzoquinone (BQ). Although the photodegradation reaction was performed under simulated solar irradiation conditions on the pristine and modified perovskites, lower amounts of the same aromatic by-products were determined by the HPLC tool. The gas phase measurements (CO_2_ time monitoring) showed that almost 16% µmoles of phenol were mineralized in the presence of Xe light over the Fe@RbLTO catalyst, albeit only 8% µmoles of CO_2_ were produced over the original RbLTO. In these circumstances, the TiO_2_ Merck reference exhibited an excellent photocatalytic response, reaching ≈ 24% µmoles of CO_2_ after 3 h of Xe lamp exposure. The data collected during the simulated solar light experiments show a lower mineralization rate of phenol; however, once again, a higher value for the Fe^2+^-modified sample can be noticed, relative to pristine RbLTO. After the stability test of Fe@RbLTO for a 10 h reaction time (Xe lamp-source irradiation), it can be observed that a higher amount of CO_2_ was produced (≈23% µmoles) (Supplementary Materials, Figure S1). This is due to the multiple-step transformation of the intermediates, which finally lead to the mineralization of phenol.

**Figure 10:**
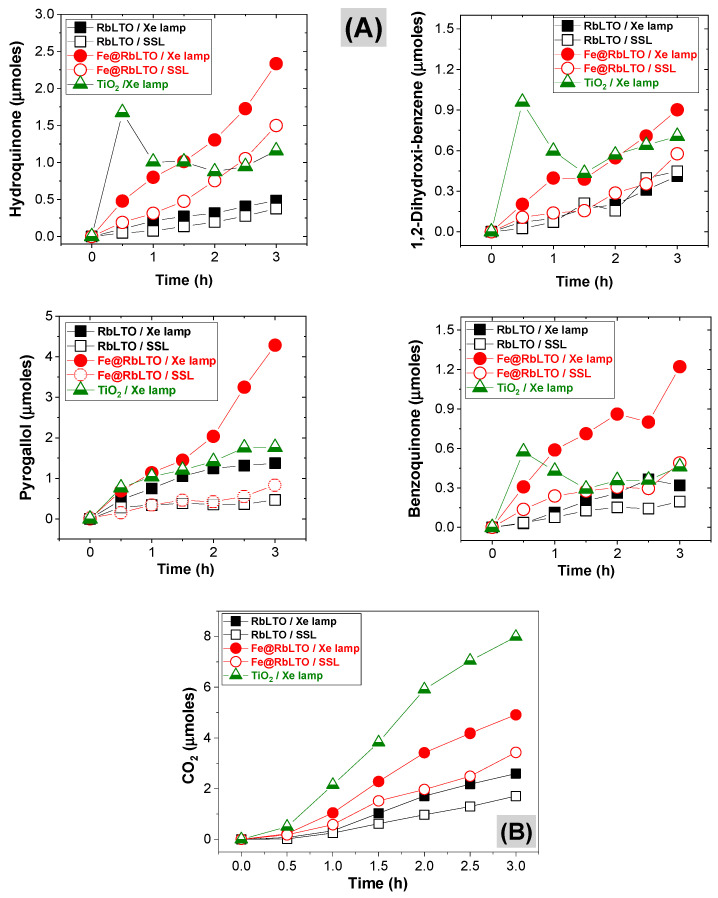
Time course of (**A**) aromatic by-products and (**B**) CO_2_ formation during the photocatalytic oxidation of phenol over RbLTO and the Fe@RbLTO photocatalyst, tested under both UV (Xe lamp) and simulated solar light conditions (AM 1.5). TiO_2_ Merck is used as a reference and evaluated after Xe lamp exposure. Initial conditions of each run: 0.050 g of catalyst, 25 mg·L^−1^ of aqueous phenol, pH 6.5, and T = 18 °C.

Figure 10A shows the formation of by-products over time, during the photocatalytic degradation of phenol over the TiO_2_ Merck reference, RbLTO, and Fe@RbLTO samples, while Figure 10B displays CO_2_ production following the reaction. After the exposure of Fe@RbLTO to the Xe lamp, the formation of the hydroxylated by-products (e.g., HQ, DHBZ, and PYROG) took place and gradually increased over time. At the same time, benzoquinone is formed during the reaction. The same behavior was perceived for the pristine RbLTO. When the samples are exposed to simulated solar irradiation, both photocatalysts show the formation of the same intermediate products appearing in lower quantities.

**Figure 11:**
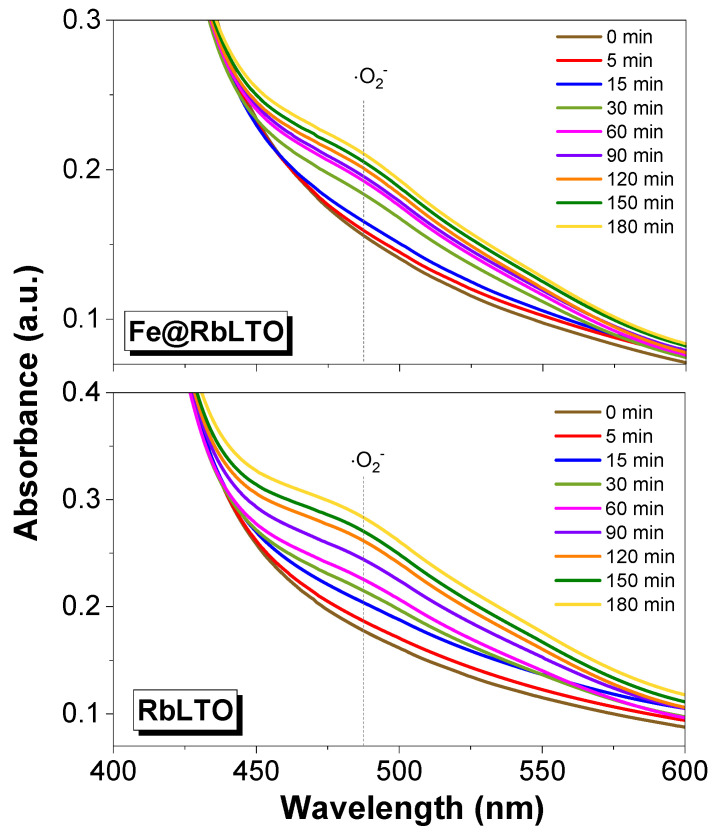
Photogeneration of superoxide anion (•O_2_^−^) over the RbLTO and Fe@RbLTO photoocatalysts.

Figure 11 displays the photogeneration of the superoxide anion (•O_2_^−^) over the RbLTO and Fe@RbLTO photocatalysts.

The ability of the catalyst powders to photogenerate •O_2_^−^ was spectroscopically checked based on the formazan characteristic peak (485 nm) resulting from the reaction of the XTT sodium salt with the O₂⁻ species produced in the reaction medium. From Figure 11, a slight difference can be perceived between the samples: the RbLTO sample suggests a higher tendency for generating O_2_⁻relative to the Fe@RbLTO sample.

## 4. Discussion

Several factors impact photocatalytic efficiency, including the amount of catalyst, the solution’s pH, the substrate concentration, the light intensity, and/or the catalyst composition. The published work points out that the active species involved in a photocatalytic process are the hole (h^+^), hydroxyl radicals (•OH), and superoxide anions (•O_2_^−^). Regarding the photocatalysts synthesized for this research, the following parameters were varied: (i) the concentration of the phenol solution by choosing the values of 25 and 50 ppm, (ii) the pH solution with values of 6.5 and 9.1, (iii) the light source, and (iv) the gas mixture used during the catalytic reaction (Supplementary Materials, Figure S2). Very poor photocatalytic activity was noticed in RbLTO and Fe@RbLTO materials by varying the above-stated parameters. Conversely, when oxygen gas is bubbling along with Ar gas in the reaction media, both scrutinized catalysts exhibit photocatalytic activity. In fact, molecular oxygen is a known acceptor of photogenerated electrons, thereby generating superoxide (•O_2_^−^) anions and preventing the recombination of e^−^/h^+^ pairs [43].

Brezova et al. [44] investigated the photodegradation of phenol in an oxygen-saturated TiO_2_ suspension in the presence of dissolved metal ions (Ca^2+^, Mg^2+^, Zn^2+^, Ni^2+^, Mn^2+^, Co^2+^, Cu^2+^, Cr^3+^, and Fe^3+^). The most effective systems for phenol photodegradation were determined in the presence of iron ions due to the efficient trapping of the photogenerated electron, thus reducing the electron hole recombination rate. Wang and co-workers reported an efficient photocatalyst based on CoAl-LDH/BiPO_4_, which was able to remove phenol, under UV-light irradiation [45]. These authors ascribed its enhanced photo-activity to the efficient hole transfer from BiPO_4_ to CoAl-LDH, which hindered the recombination of photogenerated charge carriers. Trapping experiments of active species conducted by the authors indicated that the superoxide anion was the main active specie capable of degrading phenol. In a study by Sahoo et al. [46], Ag@Ag_3_VO_4_/ZnCr LDH ternary heterostructures synthesized through hydrothermal and co-precipitation methods showed excellent photocatalytic activity for phenol photo-oxidation. The scavenger tests performed under simulated sunlight for the Ag@Ag_3_VO_4_/ZnCr LDH composite indicated that the •O_2_^−^ anion was the dominant reactive species, whereas h^+^ and •OH played a secondary role in the photocatalytic oxidation of phenol. Krasheninnikova et al. [47] fabricated ALn_2_Ti_2_NbO_10_ (A = Rb, H, and Ln = Pr, Nd)-layered perovskites for the photocatalytic oxidation of methylene blue (MB) under UV light. Their work revealed that only •O_2_^−^ species could effectively oxidize the MB substrate, while hydroxyl radicals were not involved. Similar behavior was reported for RbTe_1.5_W_0.5_O_6_ pyrochlore-type photocatalysts [12] for an identical reaction. Even though hydroxyl radicals were generated during the photocatalytic process, the authors admitted that •O_2_^−^ was the main oxidative agent. Our previous study on the photocatalytic degradation of phenol (Ph) over pristine and noble metal-modified TiO_2_ demonstrated that O_2_^−^ oxidizes mildly phenol to oxygenated products (hydroquinone, benzoquinone, and catechol). In a parallel process, hydroxyl radicals, yielded by TiO_2_, mineralize phenol into a CO_2_ end product [35].

The main photocatalytic processes that take place over the layered perovskite-based photocatalysts are represented by the following equations (Equations (1)–(6)) [48]:

Layered perovskite + hν → e^−^ + h^+^ (1)

O_2_ + e^−^ → •O_2_^−^ (*weak oxidant*) (2)

•O_2_^−^ + H^+^ → HO_2_^−^ (3)

HO_2_^−^ + H^+^ + e^−^ → H_2_O_2_ (4)

H_2_O_2_ → 2•OH (*strong oxidant*) (5)

Phenol +•OH → CO_2_ + H_2_O (6)

Based on the characterization and photocatalytic data from this study, it can conclusively be said that, by adding molecular oxygen into the reaction media, a superoxide (•O_2_^−^) anion can be generated, which is a weak oxidant [49]. As a result of the increased •O_2_^−^ yield, greater organic pollutant degradation rates may be attained. In fact, the photocatalytic degradation of phenol went through the formation of hydroxylated intermediates (hydroquinone, catechol, and pyrogallol) (Equation (7)). Also, benzoquinone was formed during the degradation process (Equation (8)).

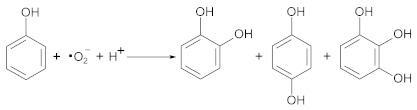
(7)

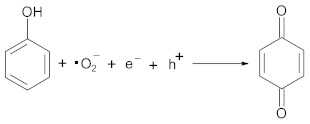
(8)

The existence of iron species over the layered perovskite compound led to enhanced visible-light absorption, a narrower band gap, and promoted the effective separation and transfer of photogenerated charge carriers, thus enhancing the photocatalytic performances. In this vein, one can explain the improved photocatalytic activity of Fe@RbLTO compared to RbLTO-layered perovskites.

In summary, synthesized, layered perovskites can be effectively employed for the reaction of phenol photodegradation under Xe light/simulated solar irradiation by generating reactive oxygen species that allow the efficient removal of the pollutant.

## 5. Conclusions

An RbLaTa_2_O_7_-layered perovskite was successfully modified by FeCl_2_ through a facile molten flux approach, maintaining its lamellar-like morphology. The catalytic efficiency of the pristine and Fe^2+^-modified perovskites was studied for the photocatalytic degradation of phenol using both an Xe lamp and solar simulator as irradiation sources.

XRD analysis confirmed the layered perovskite phase with intense reflections specific to RbLaTa_2_O_7_ Dion–Jacobson-layered perovskite materials over both original and iron-modified solids. The SEM micrographs demonstrate that Fe@RbLTO maintains a lamellar-like morphology like the pristine sample. Fe@RbLTO had a narrow band gap of about 1.82 eV, as determined from the Tauc plot from the UV-Vis spectra. The Fe^2+^-modified layered perovskite is characterized by improved optical properties, which are highly beneficial for photocatalytic applications. H_2_-TPR experiments provided information on the existence of finely dispersed Fe^2+^ species on the surface of the layered perovskite within a certain amount inserted into the interlayer space.

The catalytic performance of Fe@RbLTO for the phenol photo-oxidation reaction expressed by conversion and selectivity is better than that of the original RbLaTa_2_O_7_.

This study demonstrates the great potential of a transition chloride-modified layered tantalate as a light-harvester photocatalyst.

## Figures and Tables

**Table 1:** The unit cell parameters, d-spacing, and crystallite size of the materials.

Sample	Unit Cell Parameters (nm)	d-Spacing, Line (001) (nm)	Crystallite Size (nm)
RbLTO	a = 0.3882 c = 1.1121	1.1185	11.4
Fe@RbLTO	a = 0.3793 c = 1.1009	1.1193	15.2

**Table 2:** The phenol conversion and pseudo-first-order rate constants for the photocatalytic reaction.

Sample	Phenol Conversion after 3 h of Reaction (%)		Constant Rate k (min^−1^)	
	Xe Lamp	SSL	Xe Lamp	SSL
RbLTO	16.5	10.7	0.066	0.041
Fe@RbLTO	43.4	21.5	0.119	0.078
TiO_2_ Merck	37.2	-	0.194	-

## Data Availability

The original contributions presented in the study are included in the article/Supplementary Materials, further inquiries can be directed to the corresponding authors.

## Supplementary Materials

The following supporting information can be downloaded at: https://www.mdpi.com/article/10.3390/ma17194862/s1, Figure S1: Product distribution (% µmoles) after 10 h of degradation of phenol over the Fe@RbLTO under Xe light irradiation. Reaction conditions: 0.050 g of catalyst, 25 mg·L−1 of phenol, pH 6.5, T = 18 °C; Figure S2: Variation of the reaction conditions over RbLTO and Fe@RbLTO catalysts for the photocatalytic degradation of phenol (0.050 g of catalyst, T = 18 °C).
