# Supplementary material for: Improved Photocatalytic Activity of Dion–Jacobson-Type Tantalate Perovskites Modified with FeCl2
Source: Materials (Basel). 2024 Oct 2;17(19):4862. doi: 10.3390/ma17194862 (PMC11477869; doi:10.3390/ma17194862)
Supplement: Supplementary file 1 [file materials-17-04862-s001.zip › materials-3164844-supplementary.pdf]

Supplementary Materials

Improved Photocatalytic Activity of Dion–Jacobson-Type Tantalate Perovskites Modified with $FeCl_2$

Monica Pavel *, Crina Anastasescu, Irina Atkinson, Florica Papa and Ioan Balint *

"Ilie Murgulescu" Institute of Physical Chemistry of the Romanian Academy, 202 Spl. Independentei, 060021 Bucharest, Romania

* Correspondence: mpavel@icf.ro (M.P.); ibalint@icf.ro (I.B.)

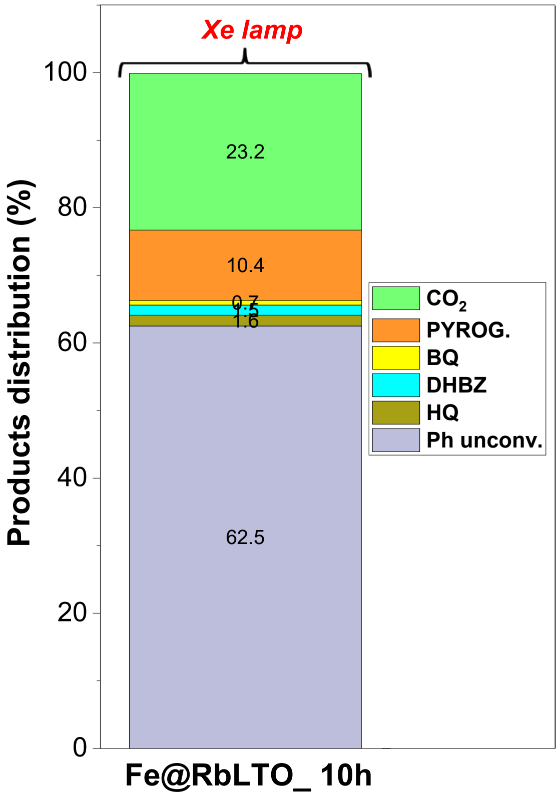

Figure S1. Product distribution (% µmoles) after 10 h of degradation of phenol over Fe@RbLTO under Xe light irradiation. Reaction conditions: 0.050 g of catalyst, 25 mg•L^{-1} phenol, pH 6.5, T = 18 °C.

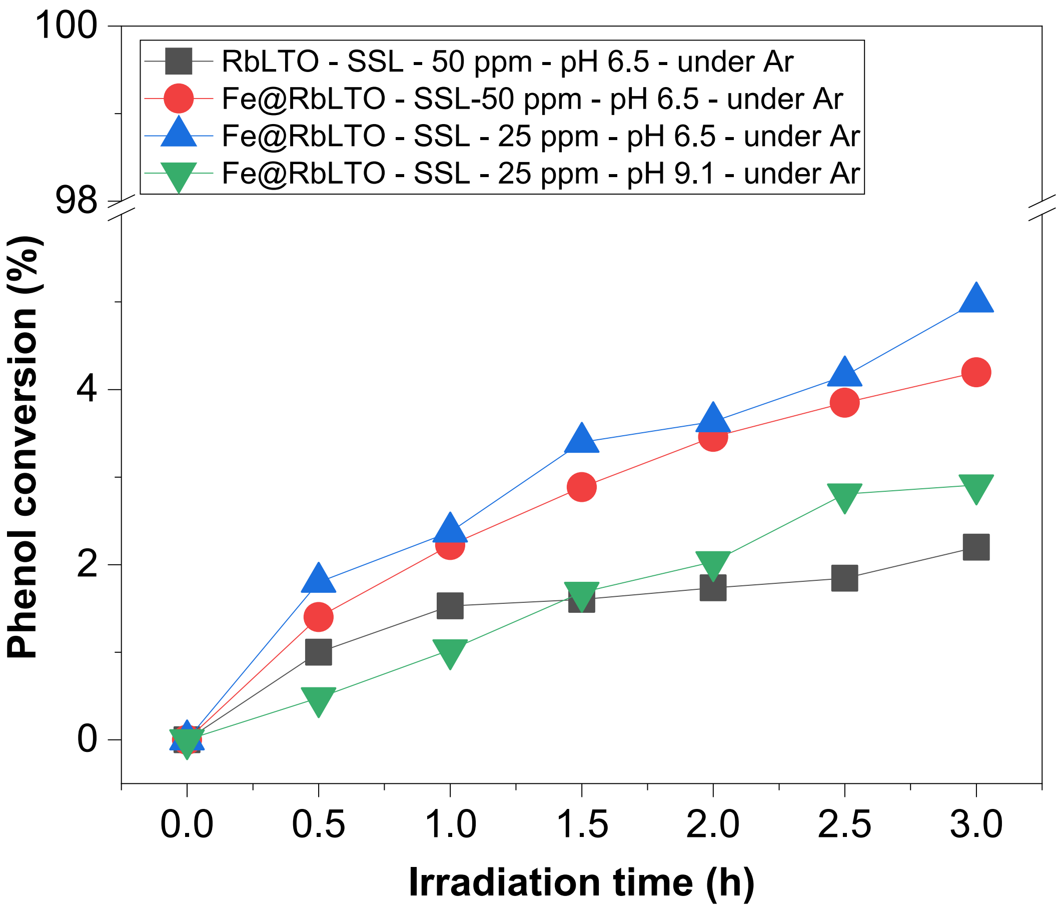

Figure S2. Variation of the reaction conditions over RbLTO and Fe@RbLTO catalysts for the photocatalytic degradation of phenol (0.050 g of catalyst, T = 18 °C).
